# In Silico Evidence of the Multifunctional Features of *Lactiplantibacillus pentosus* LPG1, a Natural Fermenting Agent Isolated from Table Olive Biofilms

**DOI:** 10.3390/foods12050938

**Published:** 2023-02-22

**Authors:** Elio López-García, Antonio Benítez-Cabello, Javier Ramiro-García, Victor Ladero, Francisco Noé Arroyo-López

**Affiliations:** 1 Food Biotechnology Department, Instituto de la Grasa (CSIC), Carretera Utrera Km 1, Campus Universitario Pablo de Olavide, 41013 Seville, Spain; 2 Technology and Biotechnology Department, Instituto de Productos Lácteos de Asturias (IPLA-CSIC), Paseo Río Linares s/n, 33300 Villaviciosa, Spain

**Keywords:** probiotic, starter culture, genome overview, fermented vegetables, whole-genome sequencing

## Abstract

In recent years, there has been a growing interest in obtaining probiotic bacteria from plant origins. This is the case of *Lactiplantibacillus pentosus LPG1*, a lactic acid bacterial strain isolated from table olive biofilms with proven multifunctional features. In this work, we have sequenced and closed the complete genome of *L. pentosus LPG1* using both Illumina and PacBio technologies. Our intention is to carry out a comprehensive bioinformatics analysis and whole-genome annotation for a further complete evaluation of the safety and functionality of this microorganism. The chromosomic genome had a size of 3,619,252 bp, with a GC (Guanine-Citosine) content of 46.34%. *L. pentosus LPG1* also had two plasmids, designated as pl1LPG1 and pl2LPG1, with lengths of 72,578 and 8713 bp (base pair), respectively. Genome annotation revealed that the sequenced genome consisted of 3345 coding genes and 89 non-coding sequences (73 tRNA and 16 rRNA genes). Taxonomy was confirmed by Average Nucleotide Identity analysis, which grouped *L. pentosus LPG1* with other sequenced *L. pentosus* genomes. Moreover, the pan-genome analysis showed that *L. pentosus* LPG1 was closely related to the *L. pentosus* strains *IG8*, *IG9*, *IG11*, and *IG12*, all of which were isolated from table olive biofilms. Resistome analysis reported the absence of antibiotic resistance genes, whilst PathogenFinder tool classified the strain as a non-human pathogen. Finally, in silico analysis of *L. pentosus LPG1* showed that many of its previously reported technological and probiotic phenotypes corresponded with the presence of functional genes. In light of these results, we can conclude that *L. pentosus LPG1* is a safe microorganism and a potential human probiotic with a plant origin and application as a starter culture for vegetable fermentations.

## 1. Introduction

*Lactobacillales*, commonly called lactic acid bacteria (LAB), are an order of Gram-positive and acid-tolerant bacteria that produce lactic acid as the major metabolic end product obtained from carbohydrate fermentation. Their large genetic versatility allows them to colonize a wide range of ecological niches, ranging from the mammalian gut microbiota to a large number of fermented foods [1]. This is the case of table olives, one of the most important fermented vegetables in Mediterranean countries, with a worldwide production that exceeds 3 million tonnes/year [2]. *Lactiplantibacillus pentosus* and *Lactiplantibacillus plantarum* (formerly known as *Lactobacillus pentosus* and *Lactobacillus plantarum*), together with diverse species of yeasts, are among the main microorganisms and LAB species responsible for table olive fermentation, determining the flavor, quality, and safety of the final product [3,4,5].

Traditionally, the market for probiotic foods was dominated by fermented dairy products. However, in recent years, the demand for vegetal-based probiotic products has grown due to a shift in consumer preference toward healthier alternatives. In addition, vegetarian activism and lactose intolerance have driven the search for new dairy-free and vegan products. Therefore, the emergence of new vegetal-based probiotics should be encouraged to satisfy societal demand, contributing to the rapidly growing global market of approximately 50 billion USD [6]. 

In recent years, table olives have emerged as a solid alternative to dairy products as a carrier of beneficial microorganisms to consumers. Certain LAB strains have the ability to form biofilms on the olive epidermis, obtaining fermented olives with more than 10 million UFC/g [7]. *L. pentosus LPG1* (hereinafter *LPG1*) is a fermentation agent obtained from table olive biofilms with proven probiotic and technological features based on previous in vitro and in vivo studies [8,9]. Thereby, *LPG1* has shown anti-inflammatory, esterase, and phytase activities, a reduction in cholesterol levels, lactic acid production, and the inhibition of food-borne pathogens. It has also shown the ability to adhere to Caco-2 cells, as well as the absence of antibiotic resistance and hemolytic activity, among other features. However, the precise genomic mechanisms involving its functions are still unknown. Advances in genome sequencing techniques together with cost reduction have allowed an advancement of the knowledge, not only of the probiotic potential of the strains, but also of the technological potential. In recent years, several genomes of *L. pentosus* isolated from table olives have been sequenced [10,11,12,13,14], as well as from other vegetal-derived matrices [15].

Our aim in the present study is to expand our knowledge of the functional and safety features of the *LPG1* strain. For this purpose, we have sequenced, annotated, and closed its full genome, including chromosome and plasmids, correlating the technological and probiotic features with the presence of functional genes after in silico analysis. Our work was performed under the recommendations of EFSA, which requires full genome sequencing and annotation of novel strains that are intended for biotechnological applications [16].

## 2. Materials and Methods

### 2.1. Bacterial Strain, Culture Conditions, and DNA Isolation

The *LPG1* strain was previously isolated from Spanish-style table olive biofilms and identified by molecular methods [8]. *LPG1* was grown in Man Rogosa and Sharpe agar (Oxoid, Basingstoke, UK) at 37 °C for 48 h prior to DNA isolation. Then, genomic and plasmid DNA was extracted and purified using the protocol described by Martín-Platero et al. [17]. The integrity of the extracted DNA was confirmed by visualization in agarose gel (0.9%), while its concentration was determined by a Qubit 4 fluorometer, obtaining a DNA concentration of 319 ng/µL.

### 2.2. Genome Sequencing, Assembly, and Annotation

Whole-genome sequencing of *LPG1* was carried out using two platforms, Illumina HiSeq (Eurofins, Luxemburg) and PacBio (FISABIO, Valencia, Spain). The raw reads obtained from both methodologies were curated by Trim Galore and the fastp tool, respectively. Then, the Illumina and PacBio reads were checked for quality control by FastQC and the MultiQC tool, respectively.

A hybrid assembly was performed using Unicycler a recent assembler for bacterial genomes from a combination of short and long reads [18]. Finally, the genome annotation of the *LPG1* strain was done using the Prokaryotic Genome Annotation System (Prokka) version 1.14.6 [19]. A circular genomic map was obtained through GView.js software [20]. Genomic data were deposited in the European Nucleotide Archive under Bioproject number PRJEB51357.

### 2.3. Phylogenetic and Pan-Genome Analysis

To verify the taxonomic identity of the *LPG1* strain, an analysis of the Average Nucleotide Identity (ANI) was performed that included different strains of *L. plantarum*, *L. paraplantarum*, *L. pentosus*, and other genera from the *Lactobacillaceae* family (Table S1, Supplementary Materials). ANI analysis was performed using the JSpecies Web Server after plasmid removal [21]. Clustering and heatmap were performed by using HemI version 2.0 [22]. Moreover, a total of 62 fully assembled genomes of *L. pentosus* strains were retrieved from the NCBI GenBank (https://www.ncbi.nlm.nih.gov/genbank/) (accessed on 10 July 2022) for pan-genome analysis (Table S2, Supplementary Materials). All genomes were re-annotated with the same tool (Prokka) to avoid biases of the annotation process in the comparisons due to different annotation methods. The output general feature format (GFF) was used to perform a pan-genome analysis to identify core, accessory, and unique genes using Roary by setting a threshold of 95% BLASTp [23]. Thus, four different classes of genes belonging to the core (99% < strain < 100%), soft-core (95% < strain < 99%), shell (15% < strain < 95%), and cloud (0% < strain < 15%) groups were obtained.

### 2.4. Food Safety Assessment

The genome safety of *LPG1* was assessed using several bioinformatics tools. First, the Comprehensive Antibiotic Resistance Database (CARD) and Resistance Gene Identifier (RGI) tool were used to identify antibiotic resistance genes [24]. Second, acquired antimicrobial resistance (AMR) genes were screened by ResFinder software version 4.1, with a threshold of 80% identity and 60% for minimum length [25,26,27]. In addition, PathogenFinder 1.1 and VirulenceFinder 2.0 were employed to predict the bacteria pathogenicity towards human hosts and the identification of acquired virulence genes, respectively [28,29,30].

### 2.5. Technological and Probiotic Genomic Assessment

Functional classification of genes into COGs (Cluster of Orthologous Groups) was performed by EggNOGmapper (version 2.0), a tool for functional annotation based on precomputed orthology assignments from the EggNOG database (version 5.0) [31]. Furthermore, KofamKOALA (version 2.2) was employed for the Kyoto Encyclopedia of Genes and Genomes Orthology (KEGG) assignment and mapping of the metabolic pathways [32]. Moreover, for the automated annotation of Carbohydrate-active enzymes (CAZymes), the dbCAN2 metaserver was used, which integrates three search tools, an HMMER search against the dbCAN HMM database, a DIAMOND search against the CAZy database, and an HMMER search against dbCAN-sub, a database of carbohydrate-active enzyme subfamilies for substrate annotation [33,34]. The annotation of CAZymes was considered valid when it was consistent across at least two tools.

The genes clusters coding bacteriocins and ribosomal-synthesized and post-translationally modified peptides (RiPPs) were predicted by Bagel Database through Bagel4 and the antiSMASH version 6.1.1 web server tool [35,36]. 

The prediction of probiotic and technological genes in *LPG1* was carried out by searching the National Center for Biotechnology Information (NCBI). The whole *LPG1* genome was subjected to BLAST from the amino acid sequences of genes of interest using BLASTp, with identity percentage, E-value, and query percentage coverage threshold values of 70%, 1E-20 and 70%, respectively. Subsequently, the gene resulting from the BLAST, and not annotated by the Prokka and eggNOG annotation method, was exposed to a new BLAST search in UniProt, as many entries in this database are manually reviewed by experts. Table S3 (Supplementary Materials) shows the list of genes with technological and probiotic potential studied in the present study.

### 2.6. Identification of Prophage, Genomic Island, and Other Insertion Sequences

The prophage regions were predicted using Phage Search Tool Enhanced Release (PHASTER) [37,38]. Thus, the length, localization, GC content, and gene annotation for each prophage were predicted. The predicted intact prophages were compared to the Virus-Host DB database, and a proteomics tree of the viral genome sequence was generated through the VIPtree tool, which was based on the genome-wide sequence similarities calculated by tBLASTx [39,40]. Moreover, the genomic islands (GI) were searched against several databases using IslandViewer 4 [41]. In addition, for the search of integrons, we used the software Integronfinder [42]. Finally, the ISFinder database and ISsaga tool were used for the annotation of transposase and mobile elements [43,44].

### 2.7. Analysis of CRISPR−Cas Sequences

Coding sequences for Clustered Regularly Interspaced Short Palindromic Repeats (CRISPR) and CRISPR-associated genes (Cas) were scanned using CRISPRCasFinder v.1.1.2-I2BC [45,46]. Then, CRISPR arrays were confirmed according to the CRISPRdb database.

## 3. Results and Discussion

### 3.1. Genome Features

The genome of LAB contains mobile elements and repeat regions, which makes it difficult to close the full genome using exclusively short-read sequences obtained from the Illumina platform. For this reason, the *LPG1* genome was sequenced and closed in this study by the hybrid assembly of the sequences obtained from both Illumina (short reads) and PacBio (long reads) technologies.

The *LPG1* complete genome consisted of 3,700,533 bp with a G+C content of 46.23%. This strain had a unique and circular chromosome of 3,619,252 bp with a G+C content of 46.34%, and two sequenced plasmids, designated as pl1LPG1 and pl2LPG1, with lengths of 72,578 and 8713 bp, respectively. The sequence blast results revealed that the plasmids pl1LPG1 and pl2LPG1 showed the highest similarity to the plasmid number 6 of *L. pentosus KW2* and plasmid number 10 of *L. plantarum M19*, respectively. Further genome annotation showed that the sequenced genome consisted of 3,345 coding genes and 89 non-coding sequences (73 *tRNA* and 16 *rRNA* genes). Table S2 (Supplementary Materials) shows the length and GC content for a total of 62 *L. pentosus* genomes obtained from the NCBI database. The genome sizes ranged from 3,426,320 bp (*KAC1* strain) to 4,036,510 (*KW1* strain), while the GC content ranged from 44.9% (*IG1* strain) to 46.34% (*LPG1* strain). Therefore, *LPG1* is currently the *L. pentosus* strain with the highest GC content.

The genomic size and GC content of strains can be indicative of their lifestyle and preferred environmental niches. Thereby, the *LPG1* genome size was higher than that of the other genera from the *Lactobacillaceae* family such as *Lactobacillus helveticus* (2 Mb), *Lactobacillus acidophilus* (2 Mb), or *Lacticaseibacillus casei* (3 Mb). This fact is due to the ecological flexibility of *L. pentosus* and the diversity of the ecological niches this organism can colonize. Throughout their evolution, *Lactobacillaceae* species reduced their genome size to adapt to specific ecological niches such as mammalian gut microbiota. However, free-living species such as *L. pentosus* and *L. plantarum* have larger genomes due to horizontal gene transfer through plasmids, transposons, or prophages [47,48,49].

**Figure 1:**
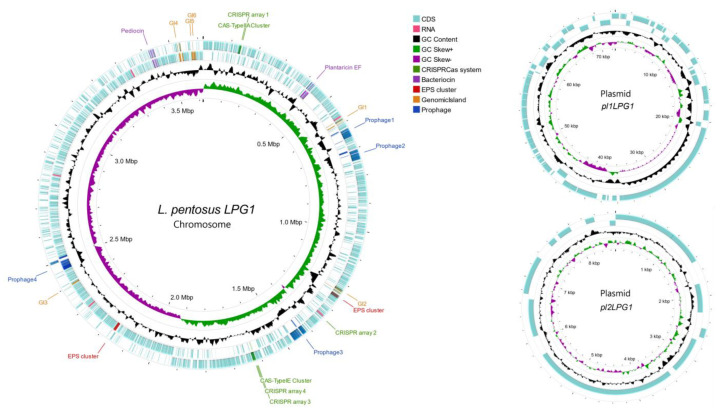
Chromosome and plasmid visualization of the *LPG1* genome. The outermost and the second ring illustrate the CDS and important markers (labels) in the forward strand and reverse strand respectively; the third circle (black) presents the GC content, the following ring denotes the GC skew index, and the last innermost ring displays the genome size.

An overview of the *LPG1* chromosome and its two plasmids is shown in Figure 1. It is noteworthy that two clusters encoding bacteriocins, as well as two clusters of exopolysaccharide production, were found in the genome. Likewise, a total of four prophages and six GI were predicted to be introduced into the chromosome. Finally, two CRISPR-Cas systems were found, as well as four CRISPR arrays.

**Figure 2:**
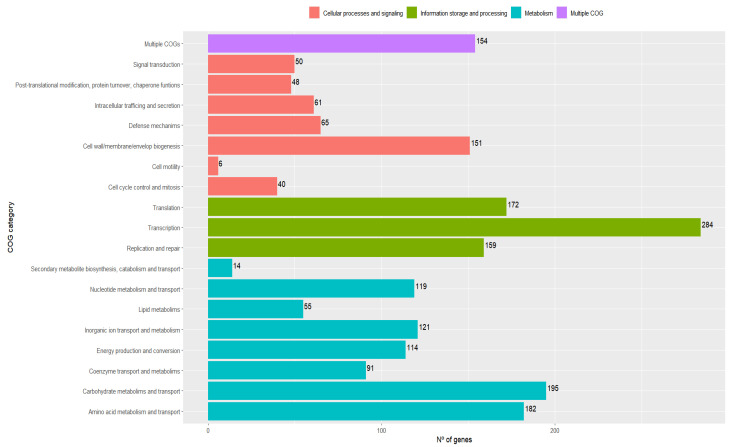
Functional classification into COG (clusters of orthologous groups) and the number of genes by categories of predicted CDSs of the LPG1 genome performed by EggNOGmapper. The genes with unknown functions and not associated with COG are not shown.

Functional classification into COG categories performed by EggNOGmapper provided an overview of the genes that *LPG1* contains. Protein-coding sequences were functionally divided into 19 COG categories; others were assigned to unknown functions or did not belong to COG. The category with the highest number of coding genes was transcription, which may be due to the genomic plasticity of the strain. Carbohydrate transport and metabolism was the second most abundant category in terms of genes coding for this function, which reflects the great capacity of *LPG1* to capture sugars and transform them, either by catabolism or by anabolism to produce more complex carbohydrates. At the opposite extreme were the low numbers of genes coding for cell motility. Figure 2 shows the number of assigned genes by COG category.

### 3.2. Phylogenetic and Comparative Analyses

**Figure 3:**
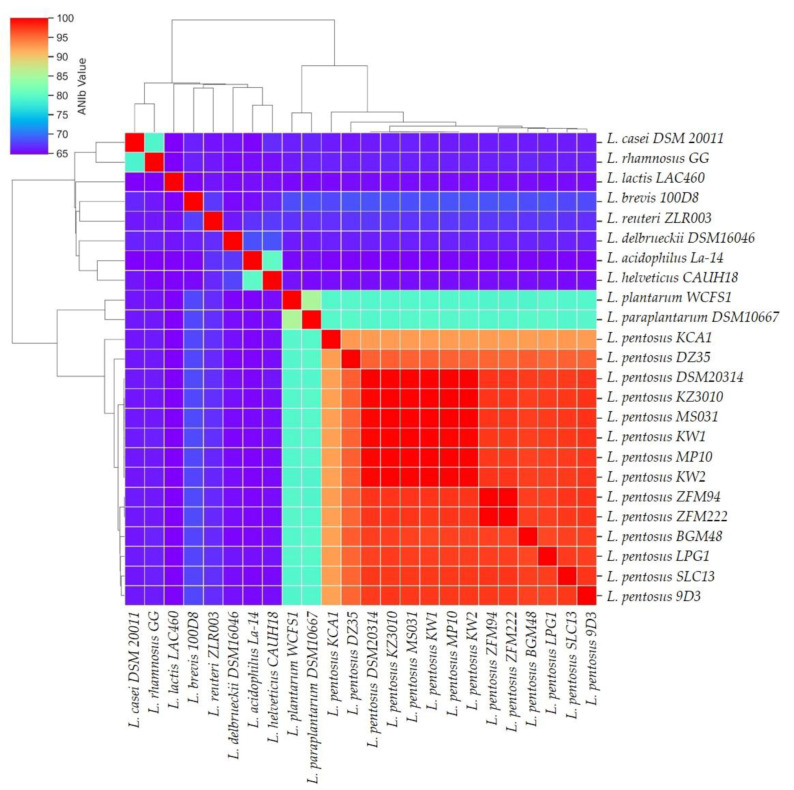
Taxonomy hierarchical clustering and heat map of ANI (average nucleotide identity) analysis included different strains of the *L. pentosus*, *L. plantarum*, and *L. paraplantarum* species, as well as other genera from the *Lactobacillaceae* family.

The complete genome assembly of *LPG1* was used to verify its taxonomy and phylogenetic relationship through ANI analysis. Studies recently showed ANI analysis as a robust measure of the genetic and evolutionary distance, as ANI analysis is based on a large number of conserved genes [50]. Moreover, phylogenetic analysis based exclusively on the *16S ribosomal* gene does not distinguish between *L. pentosus*, *L. paraplantarum* or *L. plantarum* species [51]. For this reason, in the ANI analysis, we also included different strains of *L. pentosus*, *L. plantarum*, *L. paraplantarum*, as well as other genera from the *Lactobacillaceae* family (Table S1). ANI analysis revealed that *LPG1* was grouped with the rest of the *L. pentosus* strains with >94% of the ANI value, above the threshold value to consider two organisms as the same species [52]. Moreover, *L. plantarum WCFS1* was grouped together with *L. paraplantarum DSM10667* strain in another sub-cluster, which was very different from the rest of the *Lactobacillaceae* genomes included in the present analysis (Figure 3).

**Figure 4:**
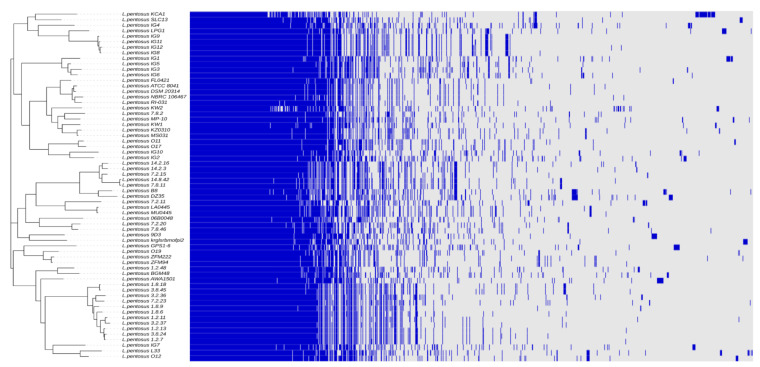
Hierarchical clustering and heat map of the gene matrix for the pan-genome analysis using the 63 *L. pentosus* strains. Blue/presence, gray/absence of the gene.

The pan-genome of *L. pentosus* was analyzed, including our *LPG1* strain as well as 62 other *L. pentosus* strains available in the NCBI database (https://www.ncbi.nlm.nih.gov/genbank/) (accessed on 10 July 2022) (Table S2, Supplementary Materials). For this purpose, we used the Roary program. The pan-genome composition consisted of 1407 core, 639 softcore, 2251 shell, and 5894 cloud genes (Table S4, Supplementary Materials). A total of 10,191 genes made up the full pan-genome; therefore, as genomes were added, the number of genes increased. The occurrence of a small number of core genes could be due to the small amount of *L. pentosus* genomes included in the analysis and to the fact that the threshold of presence in the analyzed sequences must be 99% for consideration as a core gene, which implies that if a gene is missing in any strain, it was not considered a core gene. Therefore, when the isolated strains originate from diverse environments such as fermented vegetables, female vaginas, fermented drink, soil, or fermented food, it is common for some genes to be lost. Figure 4 shows the hierarchical clustering and the heat map of the gene matrix (presence/absence) obtained after the pan-genome analysis of *L. pentosus*. As can be easily deduced from this figure, the *L. pentosus* strains more closely related to the *LPG1* strain were *IG8, IG9, IG11*, and *IG12*, all of which were isolated from table olive biofilms. 

The approach used for pan-genome analysis was also used to search probiotic marker genes (PMGs) within the shell and cloud genes present in our *LPG1* strain. Genes involved in the tolerance of bile salt, adhesion, gut persistence, response to stress, and carbohydrate metabolism were found in the *LPG1* strain in both the shell and cloud genes categories, which are also the genes shared with the rest of the *L. pentosus* strains (Table 1). Finally, from the total of 62 *L. pentosus* strains assayed, 15 strains with two complete exopolysaccharide (EPS) clusters and 48 strains with one complete EPS cluster were obtained. The genome comparison indicated the presence of only 78 *wzy* genes, which encoded the polysaccharide polymerase protein, an essential protein for EPS biosynthesis, although other crucial genes were found [53,54]. However, previous studies showed incomplete EPS clusters would likely be compensated by the genes located in other clusters [55]. Many other studies have reported the involvement of EPS in functions as diverse as biofilm formation, adhesion to the intestinal cells, inhibition of pathogen adhesion to gut cells, as well as resistance to bile salt and toxic compounds such as metal ions, and resistance under adverse conditions such as desiccation, a high salt concentration, and varying pH [56,57,58,59].

### 3.3. Food Safety Assessment

*Lactobacilli* have been commonly recognized as generally regarded as safe (GRAS) organisms due to their high safety profile. Additionally, *L. pentosus* has the qualified presumption of safety (QPS) status from the European Food Safety Agency (EFSA). However, previous studies have shown that certain *Lactobacillus* strains have antimicrobial resistance genes (AMR) that are involved in antibiotic resistance mechanisms, e.g., against chloramphenicol, tetracycline, beta-lactam, or aminoglycoside [69,70,71]. Therefore, the resistome of *LPG1* was evaluated by the RGI tool (CARD) matching only complete genes. The CARD prediction matched strict category 2 gen, *vanT* and *vanY* in the VanG cluster, which encode alanine racemase and D-alanyl-D-alanine carboxypeptidase protein, respectively. Both genes were present in a vancomycin resistance cluster. On the one hand, *vanT* converts L-serine to D-serine for peptidoglycan synthesis, and DD-carboxypeptidase catalyzes the release of D-Ala from the diacetyl-L-Lys-D-Ala-D-Ala tripeptide [72]. Of the remaining genes in the cluster, only one was predicted by the RGI tool as a loose category, the *vanS* gene in the vanG cluster. Therefore, the vanG operon was predicted as incomplete. However, previous in vitro tests showed that *LPG1* was a vancomycin-resistant strain [8]. This may be due to the presence of the D-alanine-D-alanine ligase protein encoded by the *ddlA* gene, as the *vanG* gene encodes to form the same protein, creating the dipeptide D-Ala-D-Ala, which after several transformations, develops a high affinity for vancomycin, binding it to the membrane and preventing the penetration of the antibiotic into the cytoplasm [73]. It is noteworthy that vancomycin resistance is considered intrinsic in *Lactobacilli* and not transmissible and is therefore not considered a safety concern by EFSA. As can be seen in Table S3 (pan-genome analysis) all of the *L. pentosus* strains carry both genes encoding alanine racemase and D-alanyl-D-alanine. Therefore, resistance to vancomycin is expected. On the other hand, no other *AMR* genes were obtained by the ResFinder tool for *LPG1*. These data agree with previous results obtained by Benítez-Cabello et al. [8], who described this strain as very sensitive to a wide range of antibiotics. Furthermore, no gene was predicted to be virulent based on a search in VirulenceFinder. Finally, the PathogenFinder tool classified the strain as a non-human pathogen, with a probability of being a human pathogen of only 0.075%.

### 3.4. Multifunctional Assessment

*L. pentosus* and *L. plantarum* are species typically found in a wide range of vegetable fermentations. This ability to colonize different niches is due to the presence, in both species, of an arsenal of enzymes that degrade carbohydrates released during fermentation [47,74]. 

Regarding technological features, in silico analysis of the *LPG1* strain showed a complete Embden–Meyenhorf pathway. Thereby, *LPG1* is able to ferment six-carbon carbohydrates such as glucose and fructose, both present in table olive fermentation brine. The enzymatic arsenal predicted in the annotation of the *LPG1* strain allows it to capture and hydrolyze sucrose, a compound of glucose and fructose linked through an O-glucosidic bond that is present in large quantities in table olive brine. Moreover, *LPG1* contains the mannitol degradative operon. This sugar is also common in table olive fermentations. However, in the absence of six-carbon carbohydrates, *LPG1* can ferment five-carbon sugars through the pentose phosphate pathway. Furthermore, the *LPG1* strain was predicted to contain other enzymatic tools involved in the degradation of more complex carbohydrates such as galactose, glycogen, starch, cellulose, or xylan (Table S3). 

Moreover, CAZymes analysis revealed that this enzymatic arsenal was classified into five gene classes of CAZymes. The *LPG1* genome contains 58 genes encoding the Glycosides Hydrolase family (GHs), 41 genes encoding Glucosyltransferase (GT), 3 Carbohydrates Esterases (CE), and 8 genes encoding Carbohydrate-Binding Modules (CBM). Similar results were described for other *L. pentosus* strains [65,75], which contrasts with the lower amount of CAZymes reported in other genera from the *Lactobacillaceae* family such as *L. casei*, *L. rhamnosus*, *L. acidophilus,* or *L. helveticus* [76]. This difference probably lies in the ability of *L. pentosus* to adapt to different niches and the use of diverse carbon sources as nutrients [4]. Therefore, species such as *L. rhamnosus*, *L. casei*, or *L. acidophilus* are more commonly found in host environments. In addition, to contribute to the fermentation process, the *LPG1* strain contains three copies of the gene encoding for D-Lactate dehydrogenase and three copies of the genes encoding for L-Lactate dehydrogenase, supporting the results obtained in previous studies where the *LPG1* strain was shown to be a major producer of lactic acid [8]. In these previous studies, this strain also showed high esterase activity, involved in aroma production and degradation of the bitter compound oleuropein. The subsequent in silico analysis confirmed the esterase activity, as the *LPG1* strain is predicted to contain genes encoding esterase, carboxylesterase, gallate decarboxylase, p-coumaric acid decarboxylase, and the transcriptional regulator PadR, all of which are involved in the degradation of phenolic compounds [68]. On the contrary, no gene encoding β-glucosidase was predicted, also supporting the previous results that showed the phenotype was not positive for this activity [8].

Many microorganisms produce a diverse range of products with antibiotic, antifungal, or immunosuppressant activity such as bacteriocins and Ribosomally synthesized and Post translationally modified Peptides (RiPPs), encoded by different gene clusters [77]. The occurrence of bacteriocins in fermented food is very important because this can help bacteria to inhibit the growth or eliminate foodborne pathogens and spoilage bacteria [78]. After comprehensive bioinformatics analysis, two types of bacteriocins in the *LPG1* genome were predicted; however, no RiPPs were predicted. A Pediocin gene cluster (PA-1) was identified between position 3.422.048 and 3.442.255 bp. Although bacteriocins commonly show more activity against Gram-positive bacteria, *Escherichia coli* and *Salmonella* Typhimurium have been shown to be sensitive to pediocin. Several studies have shown the relevant activity of Pediocin against *Listeria monocytogenes* [79]. In addition, the plantaricin EF gene cluster was predicted and encoded between position 420.305 and 435.375 bp. Plantaricin EF consists of two-peptide bacteriocins, and both genes were found side by side in the same operon. Both pediocin and plantaricin EF were found in other genomes of the *Lactobacillus* genus [80,81]. Previous studies showed the ability of *LPG1* to inhibit *E. coli* and *L. monocytogenes* under in vitro conditions, improving the inhibitory activity shown by the recognized probiotics strains *L. casei* Shirota and *L. rhamnosus GG* [8]. All of these data give solid reasons for the commercial use of this microorganism as a starter culture in table olives (https://www.oleica.es/productos-y-servicios/oleicastarter/, accessed on 10 July 2022) and possibly other fermented vegetables.

Regarding the probiotic potential, this activity is determined by several factors such as the capacity to survive digestion and persistence in the human gut, attachment in the intestinal cells, ability to carry out metabolic processes in a highly competitive environment, and finally to produce a beneficial effect on the host [60]. In silico analysis of *LPG1* predicted a large number of genes with the ability to perform all of these functions (Table S3). Firstly, the *LPG1* strain was predicted to harbor three genes encoding choloylglicine hydrolase, involved in resistance to bile salt. Although no choloylglicine hydrolase gene was annotated as the bile salt hydrolase gene (*bsh*), previous studies showed the ability of the *LPG1* strain to decrease cholesterol levels [8]. The ability of microorganisms to deconjugate bile via the production of bile salt hydrolase has been widely associated with their cholesterol-lowering potential in the prevention of hypercholesterolemia [61]. Additionally, other genes with important roles regarding resistance to bile salt were predicted. In this way, two chaperones genes encoding *clpC* and *clpE*, four genes encoding methionine sulfoxide reductase (*msrA_1*, *msrA_2*, *mrsA_3*, and *msrB*), two genes encoding histidine protein kinase (HKP) along with response regulator (RR), and two efflux systems related to the multidrug resistance transporter family encoding by *emrB_1* and *lmrA* were predicted [82,83]. 

A large number of genes involved in the response to acid stress and ultimately involved in persistence in the gut, such as the *DnaK*, *DnaJ*, *GroEL*, and *GroES* chaperones, as well as the GrpE protein, were found in the genome of the *LPG1* strain. Previous assays related an upregulated response to the acid environment and bile exposure to genes coding for these proteins [84,85]. Furthermore, a total of 16 complete PTS sugar transporter systems were annotated, reflecting an adaptative and flexible behavior in carbohydrate metabolism. More specifically, cellobiose PTS has been confirmed to play a key role in the competitive ability of *Lactobacillus* in the gut [86]. 

Regarding the ability of the *LPG1* strain to adhere to intestinal cells, a variety of related genes have been predicted. Thus, five genes encoding a mucus-binding protein (MucBP), two genes encoding a collagen binding-protein and fibronectin-binding domain-containing protein, and two genes encoding glyceraldehyde-3-phosphate dehydrogenase were predicted, all of them involved mainly in the colonization of the gut [62,63,64,87]. In addition, others genes involved in intestinal cell adhesion such as the *strA*, *luxS*, *dltA*, or *tuf* genes were annotated in the *LPG1* genome [88,89,90,91]. In contrast, the gene encoding S-layer protein, an important protein involved in the adhesion to intestinal cells, was not predicted [62]. EPS production also plays an important role in the adhesion to the surface, as well as conferring a certain degree of protection to the bacteria themselves [92]. Moreover, bacterial EPS has been associated with the pathogen’s growth inhibition [93] and modulation of the immune system [94,95]. In this sense, up to two complete EPS clusters were predicted in the *LPG1* genome. 

Alternatively, a certain degree of synergism of *LPG1* with the gut microbiota could be expected due to a large number of enzymes producers of lactic acid that were predicted, especially butyrate-producing colon bacteria that transform lactic acid into butyric acid, which has been shown to be an excellent beneficial anti-inflammatory in the gut epithelium [96,97]. Previous studies showed the ability of this strain to modulate the immune response in human and murine cell lines and a colitis murine model, highlighting its anti-inflammatory activity [9].

Finally, some species of *Lactobacillus* are known to be producers of vitamin B9, also known as folate, an essential nutrient component of the human diet, that is involved in many metabolic pathways [98]. The efficiency of DNA replication, repair, and methylation is affected by folate; therefore, high amounts of folate are required by fast-proliferating cells such as leucocytes, erythrocytes, and enterocytes [99]. The *LPG1* genome was predicted to harbor an active folate cluster including genes such as *dfrA*, *fpgS_1*, *fpgS_2*, *folP*, *folE*, *folk*, and *folB*. 

### 3.5. Identification of Mobile Genetic Elements

**Figure 5:**
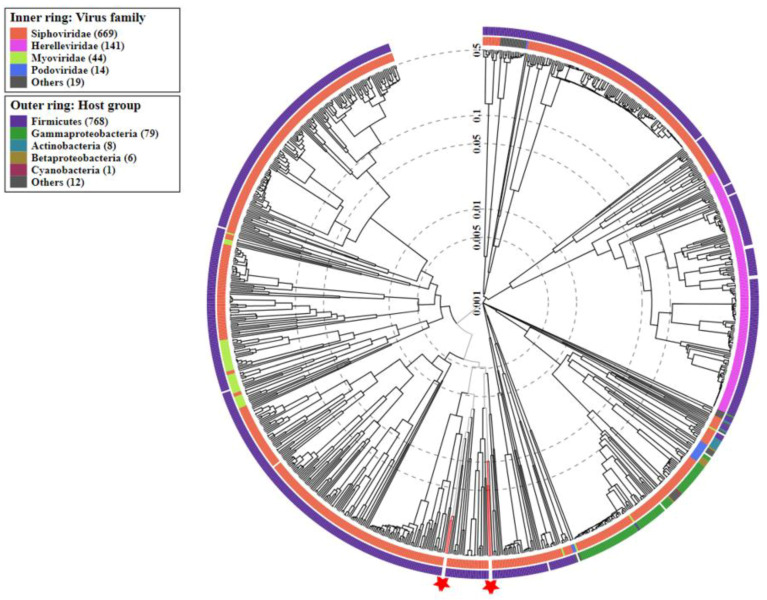
Circular proteomic tree of the viral genome sequences. The four hypothetical prophages are marked with a red star in the graph with all related viral genomes. The outermost ring presents the host group of bacteriophages; the inner ring symbolizes the virus family of phages.

Bacteriophages are viruses that infect and replicate in bacterial cells, being ubiquitous and the most abundant biological agent in the environment. Similar to most viruses, bacteriophages are species-specific or even strain-specific. When a bacteriophage infects a bacterial cell, two different strategies of replication can be developed, a lysogenic or a lytic cycle. Usually, during a lysogenic cycle, the phage genome is integrated into the bacterial chromosome, called a prophage. The prophage contains the necessary information to induce a lytic cycle, a viral reproduction method that involves the destruction of infected cells and that is usually induced in response to stress conditions [100,101]. The presence of prophages in bacterial strains with probiotic potential isolated from the fermentation process is common [102,103,104]. Furthermore, prophages are a source of new genes added to the genome, in some cases providing new features in the bacterial genomes [105,106]. These features could include antibiotic resistance genes or virulence factors [107,108]. The *LPG1* strain was predicted to contain four intact prophages according to PHASTER. A similar number of prophages have been found in other *L. pentosus* genomes [14,75,108]. The length of the prophages ranged from 39.4 Kb to 62.5 kB, and each prophage encoded about 50 proteins. Integrase was present in all prophages. Among genes predicted in the prophages sequence, no genes involved in the drug resistance mechanisms or virulence factors were detected. A comparison of the database and the proteomic tree was performed for each latent phage (Figure 5). The prophages analyzed were related to the *Siphoviridae* family, the main family responsible for infecting the *Lactiplantibacillus* genus along with the *Myoviridae* family [109].

The IslandViewer 4 tool was used to predict GI matched with whole prophages predicted through PHASTER. GI refer to discrete DNA segments that establish horizontally transferred genes in a population and frequently encode functions such as pathogenesis, symbiosis, and adaptation [110,111]. Six putative GI in the *LPG1* genome were predicted by at least one of the following methods: IslandPick, SIGI-HMM, and IslandPath-DIMOB [112,113,114]. Some of the most interesting genes found in GI of the *LPG1* strain were involved in polysaccharide biosynthesis, teichoic acid synthesis, metal ion transporters, and carbohydrate metabolism, such as beta-galactosidase and extracellular alpha-L-arabinofuranosidase. These findings showed similarities with other *Lactobacillus* where GI included genes coding for sugar metabolism and transport and exopolysaccharide biosynthesis [115,116]. Furthermore, a search for integrons was carried out using IntegronFinder, and no results were obtained for the presence of integrons. Perhaps the most important mobile genetic elements are the integrons, which play a key role in the dissemination of antibiotic-resistance genes [117]. 

**Figure 6:**
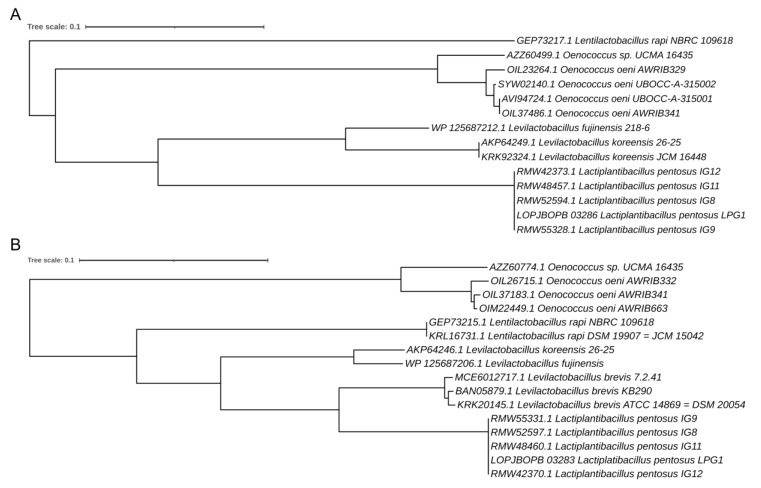
Phylogenetic analysis performed by the neighbor-joining method with 1000 replications in the bootstrap test. (**A**) Tree based on the L-arabinofuranosidase gene. (**B**) Tree based on the beta-galactosidase gene.

The GI coding for L-arabinofuranosidase and beta-galactosidase, both found within the shell genes in the pan-genome analysis, were used to find the most likely bacterium that transferred these genes to *LPG1*. Thus, BLAST-p was performed for both genes, prior to transformation into proteins. Later, the alignment was held with the sequences of most similar genomes found in the BLAST-p results. The phylogenetic tree showed that *Levilactobacillus* was probably the genus responsible for the transfer of the GI to *LPG1* (Figure 6). Interestingly, species of the *Levilactobacillus* genus have been also identified in Sicilian and Algerian table olive processing [118,119], with both species sharing the same habitats. In addition, *L. pentosus IG8, IG9, IG10*, and *IG11,* all of them from table olive processing [13], contain these genes in common with *LPG1*. Therefore, it is likely that the *LPG1* strain shares a common ancestor with all of them. These results agree with the hierarchical clustering obtained from the pan-genome analysis.

Finally, other mobile genetic elements such as the insertion sequence (IS) could play a role in gene disruption or activation due to promoter interaction to contribute to the genome plasticity [120]. Functional gene loss occurs in genomes with a large number of IS elements. Furthermore, IS elements with transposase genes flanked by two inverted repeats have the skill to move through transposition, causing DNA rearrangement [121,122]. Seventeen transposases were found in the chromosome of *LPG1*, and 13 were located in pl1LPg1, the most abundant plasmid in the *LPG1* strain. A similar number was found in other *Lactobacillus* strains such as *L. pentosus DSM20314* or *L. reuteri PNW1* [67]. However, the number was lower than that of other *L. pentosus* with putative probiotic potential such as *L. pentosus MP-10* or *L. pentosus CF2-10N* [14,123]. This could mean that the *LPG1* strain has higher transcription stability than other genomes with a large amount of IS. The transposase included 10 different families, highlighting the ISL3, IS5, and IS30 families with multiple copies.

### 3.6. Identification of CRISPR−Cas Sequences

CRISPR-Cas systems are an adaptive immune system present in prokaryotic organisms, both bacteria, and Archaea, conferring resistance to exogenous genetic material, whether from pathogens, other bacteria, phages, or any element that could lead to a risk to the organism at the expression levels [124,125]. Previous studies have shown the occurrence of the prophages was lower in strains with the CRISPR-Cas system than in strains without this immune system, suggesting that CRISPR-Cas systems play a key role in preventing phage infection and consequently avoiding prophage integration into the genome [100]. In the *LPG1* genome, two CRISPR-Cas systems were identified, CRISPR-Cas type I-E and type II-A. CRISPR-Cas operon type I-E and type II-A were formed by eight *Cas* genes (*cas1*, *cas2*, *cas3*, *cse1*, *cse2*, *cas7*, *cas5*, and *cas6*) and four *Cas* genes (*cas9*, *cas1*, *cas2*, and *csn2*), respectively. These results were very similar to those found in other *L. pentosus* strains such as *KCA1*, *MP-10*, *IG4*, *IG7*, *IG8*, *IG9*, *IG11*, or *IG12* [12,13,65,66]. Up to six CRISPR arrays were predicted by the CRISPRcasFinder tool but, only four were confirmed by CRISPRdb. A CRISPR array is composed of repeated elements and spacers, where spacer sequences are fragments of foreign DNA originating from plasmids, phages, or other exogenous genetic material that are incorporated into the host [125]. Therefore, the occurrence of the CRISPR-Cas system could prevent the *LPG1* strain from acquiring antimicrobial resistance genes or virulence or pathogenic factors through the horizontal transfer of genes.

## 4. Conclusions

Comprehensive bioinformatic analysis of the *LPG1* genome has revealed the presence of a large number of genes related to multifunctional features, which could be allied with many of its technological and probiotic phenotypes obtained in previous studies. A safety assessment of the *LPG1* genome also showed the absence of virulence and antibiotic resistance genes, as well as the presence of gene systems that could prevent their acquisition (CRISPR-Cas system). Conclusively, *LPG1* is a safe microorganism with great potential for use as a human probiotic from plant origin or as a starter culture for vegetable fermentations. 

## Figures and Tables

**Table 1:** Shell and cloud genes found in the *LPG1* genome related to its probiotic and technological potential.

Function Involved	Gene	Locus	Annotation	Presence in Pangenome (%)	Reference
Acid stress resistance and tolerance bile salt	*SecB_1*	LOPJBOPB_01128	Protein-export chaperone SecB	40	[60]
	*secB_2*	LOPJBOPB_01741	Protein-export chaperone SecB	79	[60]
	*oppA_2*	LOPJBOPB_00084	Peptide ABC transporter substrate-binding protein	87	[60]
	*pva2*	LOPJBOPB_03149	Choloylglycine hydrolase	68	[61]
	*lmrA*	LOPJBOPB_02793	Multidrug resistance ABC transporter ATP-binding and permease protein	40	[59]
	*emrB_1*	LOPJBOPB_00043	Multidrug efflux MFS transporter	24	[59]
	*speF*	LOPJBOPB_00263	Inducible ornithine decarboxylase	16	[60]
Adhesion	*LOPJBOPB_02921*	LOPJBOPB_02921	MucBP domain-containing protein	59	[62,63]
	*LNFPBAJA_00018*	LNFPBAJA_00018	MucBP domain-containing protein	37	[62,63]
	*LOPJBOPB_01179*	LOPJBOPB_01179	LPXTG cell wall anchor domain-containing protein	24	[60]
	*gap_1*	LOPJBOPB_00266	Glyceraldehyde-3-phosphate dehydrogenase	11	[64]
Carbohydrate metabolism	*glgP*	LOPJBOPB_00022	Glycogen phosphorylase	81	[65,66]
	*malS*	LOPJBOPB_00166	Alpha-1,4-glucan:maltose-1-phosphate maltosyltransferase	57	[65]
	*mal_1*	LOPJBOPB_00161	Oligo-1,6-glucosidase/alpha-amylase	57	[65,67]
	*iolI*	LOPJBOPB_03268	Inosose isomerase/epimerase	37	[65]
	*treP*	LOPJBOPB_00084	Alpha-trehalose phosphorylase	25	[60]
	*ycjT*	LOPJBOPB_03255	Kojibiose phosphorylase	19	[65]
	*abf2*	LOPJBOPB_03286	Intracellular exo-alpha-L-arabinofuranosidase	8	[60]
	*bgaA*	LOPJBOPB_03283	beta-galactosidase	8	[65,66]
Response to stress	*dps_2*	NFJKPBJO_00003	DNA protection during starvation protein	10	[60,67]
Degradation phenolic compound	*LOPJBOPB_02786*	LOPJBOPB_02786	Tannase	59	[68]
	*LOPJBOPB_00821*	LOPJBOPB_00821	Carboxylesterase	81	[68]
	*LOPJBOPB_02878*	LOPJBOPB_02878	PadR family transcriptional regulator	56	[68]

## Data Availability

ENA repository access number PRJEB51357.

## Supplementary Materials

The following supporting information can be downloaded at https://www.mdpi.com/article/10.3390/foods12050938/s1, Table S1: List of *Lactobacillaceae* strains included in the present study for ANI (Average Nucleotide Identity) analysis; Table S2: List of *L. pentosus* strains used in the present study for pangenome analysis; Table S3: List of genes found in *L. pentosus LPG1* related to its probiotic and technological potentials; Table S4: Results of the pan-genome analysis, presence/absence of genes in *L. pentosus* strains included in the analysis.
